# Fecal microbiota transplantation restores dysbiosis in patients with methicillin resistant *Staphylococcus aureus* enterocolitis

**DOI:** 10.1186/s12879-015-0973-1

**Published:** 2015-07-11

**Authors:** Yao Wei, Jianfeng Gong, Weiming Zhu, Dong Guo, Lili Gu, Ning Li, Jieshou Li

**Affiliations:** Institute of General Surgery, Jinling Hospital, Medical School of Nanjing University, #305 East Zhongshan Road, Nanjing, 210002 Jiangsu Province China

**Keywords:** Fecal microbiota transplantation, *Methicillin resistant Staphylococcus aureus enterocolitis*, Vancomycin

## Abstract

**Background:**

Nosocomial *Methicillin-resistant Staphylococcus aureus* (MRSA) enteritis is rare but can be fatal unless it is detected at an early stage and treated effectively. Dysbiosis of the gut is one of the leading reasons of MRSA enteritis. Fecal microbiota transplantation (FMT) is a burgeoning treatment to rectify this imbalance. But the impact of FMT on MRSA enterocoitis is still unknown yet.

**Methods:**

A total of 5 patients diagnosed as MRSA enteritis during the early postoperative period were given vancomycin 2 g/day for 3 days and FMT for three continuous days as a standard treatment.

**Result:**

There was a 100 % clinical response rate that all the symptoms resulting from MRSA enterocolitis disappeared and MRSA in the feces eliminated clearly. The microbiota profile in feces of the patients also regained balance.

**Conclusion:**

FMT can be a preferential measure to restore the dysbiosis caused by MSRA enterocolitis.

## Background

*Methicillin-resistant Staphylococcus aureus* (MRSA) is one of the major sources of nosocomial infection. Along with the widely application of antibiotics around perioperative period, MRSA infection is increasing by years. People are the natural reservoir of MRSA but its growth and reproduction is limited by other intestinal flora [1], rarely cause enteritis. Enterocolitis caused by MRSA was first reported in Europe in 1961 and then investigators in Australia, Japan and France have reported MRSA as a cause of antibiotic-associated diarrhea among hospitalized patients, but all of the reported cases were treated by vancomycin only and had no data about the changes of the gut flora [2].

Fecal microbial transplantation (FMT),infusion of fecal preparation from a healthy donor into the GI tract of a patient is being proposed as a novel therapeutic approach to modulate diseases associated with pathological imbalances within the resident microbiota, termed dysbiosis [3]. It has been used to treat intestinal disease such as inflammatory bowel diseases and *Clostridium difficile* infection, but no reports are available on its role in treating MRSA enteritis yet. Here we reported five cases of MSRA enterocolitis cured by fecal microbiota transplantation (FMT) combined with vancomycin. For the first time, we reported the changes of the microbiota in the stool during the therapeutic process.

## Methods

### Patients

This study was approved by the Institutional Ethics Committee of Jinglin hosipital. Five cases from July, 2013 to February, 2014 were collected in Jinling hospital. Written informed consent was obtained from all the patients and the donors. There were three males and two females with an age range from19–45 years (mean age, 28 years) (Table 1). No patients were on proton pump inhibitors (PPI) or had a history of MRSA prior to operation. Antibiotics given at induction and continued in the postoperative period were showed in detail (Table 2). The patients were placed on the open ward prior to development of symptoms.

**Table 1:** Basic clinical data of the patients

Patient	Underlying pathology	Operations	Ileostomy	Onset of disease after surgery
Patient 1	Crohn’s disease	The terminal ileum enterostomy	YES	2 days
Patient 2	pancreatic cancer	Whipple Procedure + Percutaneous jejunostomy	NO	4 days
Patient 3	Crohn’s disease	The terminal ileum enterostomy	YES	2 days
Patient 4	congenital intestinal malrotation	Volvulus reduction + Gastrostomy	NO	3 days
Patient 5	Crohn’s disease	The terminal ileum enterostomy	YES	4 days

**Table 2:** Drugs given at induction and continued in the postoperative period

patients	Name of drug	dose	Manufacturer
Patient 1	Vancomycin (Vancocin cp)	0.5 g q12h for 10 days	Eli Lilly and Company
Patient 2	Cefuroxime (Zinacef)	1.5 g 2 h before operation and 48 h after operation	Glaxo Wellcome Op
	Ornidazole (TUOSU)	0.5 g 2 h before operation and 48 h after operation	Sichuang Kelun Pharmaceutical Co. Ltd
	Pantoprazole (TIANLONG)	40 mg 48 h after operation	Liaoning Tianlong Pharmaceutical Co. Ltd
Patient 3	Ceftriaxone sodium Tazobactam sodium (YOUTANENG)	2 g 2 h before operation and 48 h after operation	Haikou Qili Pharmaceutical Co. Ltd
	Omeprazole (AOXIKANG)	40 mg 48 h after operation	Jiangsu Aosaikang Pharmaceutical Co. Ltd
Patient 4	Ceftriaxone sodium Tazobactam sodium (YOUTANENG)	2 g 2 h before operation and 36 h after operation	Haikou Qili Pharmaceutical Co. Ltd
	Omeprazole (AOXIKANG)	40 mg 72 h after operation	Jiangsu Aosaikang Pharmaceutical Co. Ltd
Patient 5	ceftazidime (Fortum)	2 g 2 h before operation and 72 h after operation	GlaxoSmithKline plc
	Pantoprazole (Pantoloc)	40 mg 48 h after operation	Nycomed GmbH

**Fig. 1:**
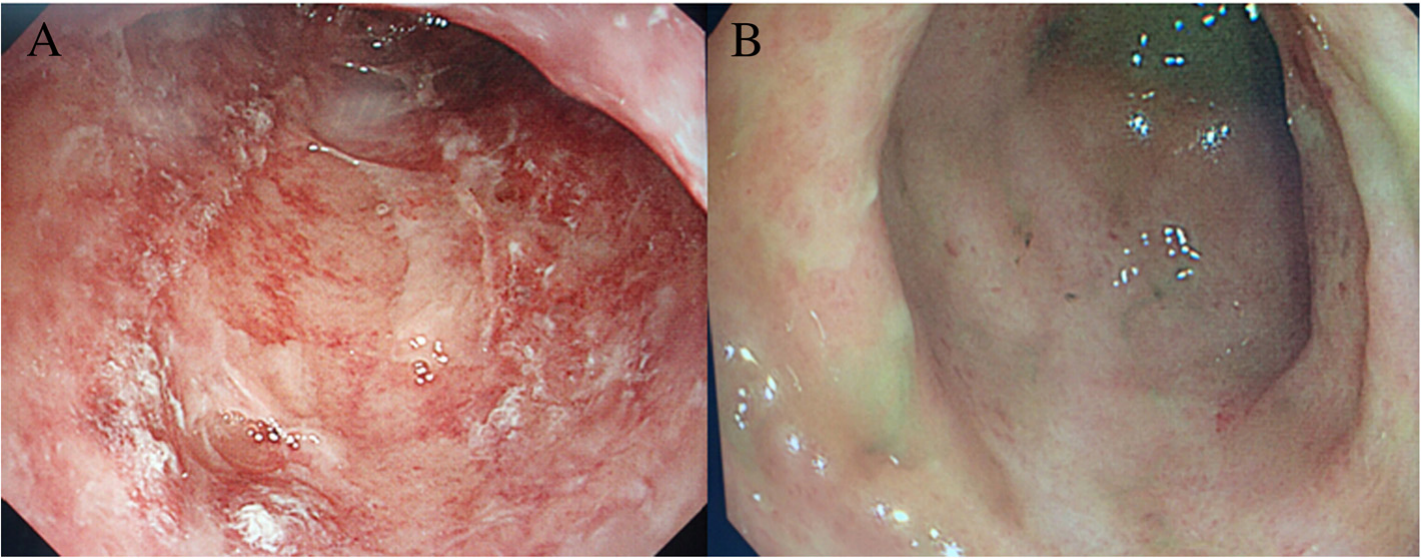
Patient 2's colonoscopy before and after treatment. **a**. showed mucosal edema and large quantity of pseudomembrane in the colon before FMT and vancomycin. **b**. the pseudomembrane disappeared and mucosa healed well after the treatment of FMT combined with vancomycin for three days. FMT = fecal microbiota transplantation

All the patients developed unexplained high fever (over 39°), bloating, nausea, vomit, a high stoma output or diarrhea in the color of yellow-green with copious amounts of mucus leading to dehydration and tachycardia after short time of operation (2-4d). Full septic screen was performed. This included bacterial swabs of stoma as well as blood, urine, ascites and gastric juice cultures. These were repeated throughout the diseased state. Fecal was investigated for *Salmonella spp.* and *Clostridia difficile*. Radiological studies included plain radiographs, ultrasonography, contrast computerized tomography (CT) to exclude intraabdominal or other infections. We got the etiology diagnosis from all the ptients’ gastric juice cultures which revealed MRSA and collected MRSA strain for further genotype identification. One patient (Patient 2) also underwent colonoscopy correspondingly showed mucosal edema and large quantity of pseudomembrane (Fig. 1).

### Donor

The intended stool donors had received no antibiotic therapy within the last 6 months. To avoid a transmission of other diseases, donors had to have a negative history for intestinal diseases or recent gastrointestinal infections, autoimmune or other immune-mediated diseases, or any kind of malignancies. Chronic hepatitis B and C, human immunodeficiency virus, and syphilis were excluded and the donors’ stool was tested for *C. difficile*, enterohemorrhagic *Escherichia coli*, *Salmonella*, *Shigella, Yersinia*, and *Campylobacter* as well as parasites. Stool samples of four donors were collected for further analysis of the fecal microbiota, unfortunately we lost a sample stool from one donor.

### Donor material preparation

Donors produced stool samples within 6 h before FMT. 60 g fresh fecal samples were blended with 350 ml sterile saline for 10 min in a designated GI laboratory space. This blended fecal mixture was then filtered through 3 gauze pieces to remove larger sediments. Filtered fecal preparation was then kept at 4 °C until FMT was performed.

### Transplantation procedure

All patients had been informed and agreed to undergo FMT. All but one (who accepted vancomycin for 10 days before FMT) were maintained on full dose of vancomycin (Vancocin cp) (500 mg, twice a day for continuous three days) until 12 h before the FMT procedure. The prepared donor microbiota was administered via nasojejunal tube once a day for 3 consecutive days (Table 3). Since the patients all suffered from severe diarrhea, no mechanical bowel preparation were done prior to FMT as usually recommended [4].

**Table 3:** Data of fecal mircobiota transplantation

Patients	Donor	Transplantation time	Transplanted stool weight	Transplantation way	Drug before transplantation
Patient 1	Unrelated Child (6 year)	One time a day for continuous three days	60 g every time	nasointestinal tube	Vancomycin^※^
					0.5 g,q12h for continuous 10 days
Patient 2	Her son (25 year)	One time a day for continuous three days	60 g every time	Jejunostomy fistula tube	Vancomycin^※^
					0.5 g,q12h for continuous three days
Patient 3	Unrelated Healthy girl (23 year)	One time a day for continuous three days	60 g every time	nasointestinal tube	Vancomycin^※^
					0.5 g,q12h for continuous three days
Patient 4	His mother (43 year)	One time a day for continuous three days	60 g every time	Gastrostomy fistula tube	Vancomycin^※^
					0.5 g,q12h for continuous three days
Patient 5	His mother (49 year)	One time a day for continuous three days	60 g every time	nasointestinal tube	Vancomycin^※^
					0.5 g,q12h for continuous three days

※ Made in Eli Lilly and Company, Trade name is Vancocin cp

### Microbial diversity and type of MRSA analysis

#### Microbial diversity analysis

Microbial DNA was extracted from fecal samples using the E.Z.N.A. ® DNA Kit (Omega Bio-tek, Norcross, GA, U.S.) according to manufacturer’s protocols. The V4-V5 region of the bacteria 16S ribosomal RNA gene were amplified by PCR (95 °C for 2 min, followed by 25 cycles at 95 °C for 30 s, 55 °C for 30 s, and 72 °C for 30 s and a final extension at 72 °C for 5 min) using primers 515 F 5’-barcode- GTGCCAGCMGCCGCGG)-3’ and 907R 5’-CCGTCAATTCMTTTRAGTTT-3’, where barcode is an eight-base sequence unique to each sample. PCR reactions were performed in triplicate 20 μL mixture containing 4 μL of 5 × FastPfu Buffer, 2 μL of 2.5 mM dNTPs, 0.8 μL of each primer (5 μM), 0.4 μL of FastPfu Polymerase, and 10 ng of template DNA. Amplicons were extracted from 2 % agarose gels and purified using the AxyPrep DNA Gel Extraction Kit (Axygen Biosciences, Union City, CA, U.S.) according to the manufacturer’s instructions and quantified using QuantiFluor™ -ST (Promega, U.S.). Purified amplicons were pooled in equimolar and paired-end sequenced (2 × 250) on an Illumina MiSeq platform according to the standard protocols. The raw reads were deposited into the NCBI Sequence Read Archive (SRA) database.

#### Type of MRSA analysis

DNA were extracted from different specimens.9 primers of SCCmecI-V were synthesized with multiple PCR technique according to the reference [5] and for STR analysis to make out the type of MRSA.

## Results

### Clinical outcome

All the patients’ MRSA enteritis was cured showing no symptoms resulting from MRSA enterocolitis. Continuous renal replacement therapy (CRRT) was performed in all patients due to severe SIRS response, and they returned to normal temperature in 2–3 days. Mean maximum stoma or stool output decreased from6426.00 ± 2707.90 ml to 1361.00 ± 951.09 ml and the mean daily fluid requirements decreased from 8505.40 ± 2071.30 ml to1706.20 ± 578.96 ml within 10 days after disease onset (Table 4). Besides, all the patients can use enteral nutrition every well without problem. They were followed up for 3 months with repeated stool culture, which all showed negative for MRSA.

**Table 4:** Clinical manifestation of the patients

patients	Temperature maximum (°C)	Maximum/minimum stoma output/stool (ml/24 h)	Maximum/minimum liquid requirements (ml/24 h)	Begin to use enteral nutrition (days after the enteritis onset)	MRSA screen
Patient 1	40.1	6970/1510	9814/1666	3 days	gastric juice
Patient 2	39.5	10840/380	10225/1230	4 days	gastric juice, abdominal drainage fluid
Patient 3	39.3	5521/2870	9800/2580	3 days	gastric juice, ascitic fluid
Patient 4	39.5	4909/770	5486/1150	2 days	gastric juice, sputum
Patient 5	40.2	3890/1275	7202/1905	2 days	gastric juice, ileum colostomy liquid

### Changes of the gut microbiota before and after FMT

**Fig. 2:**
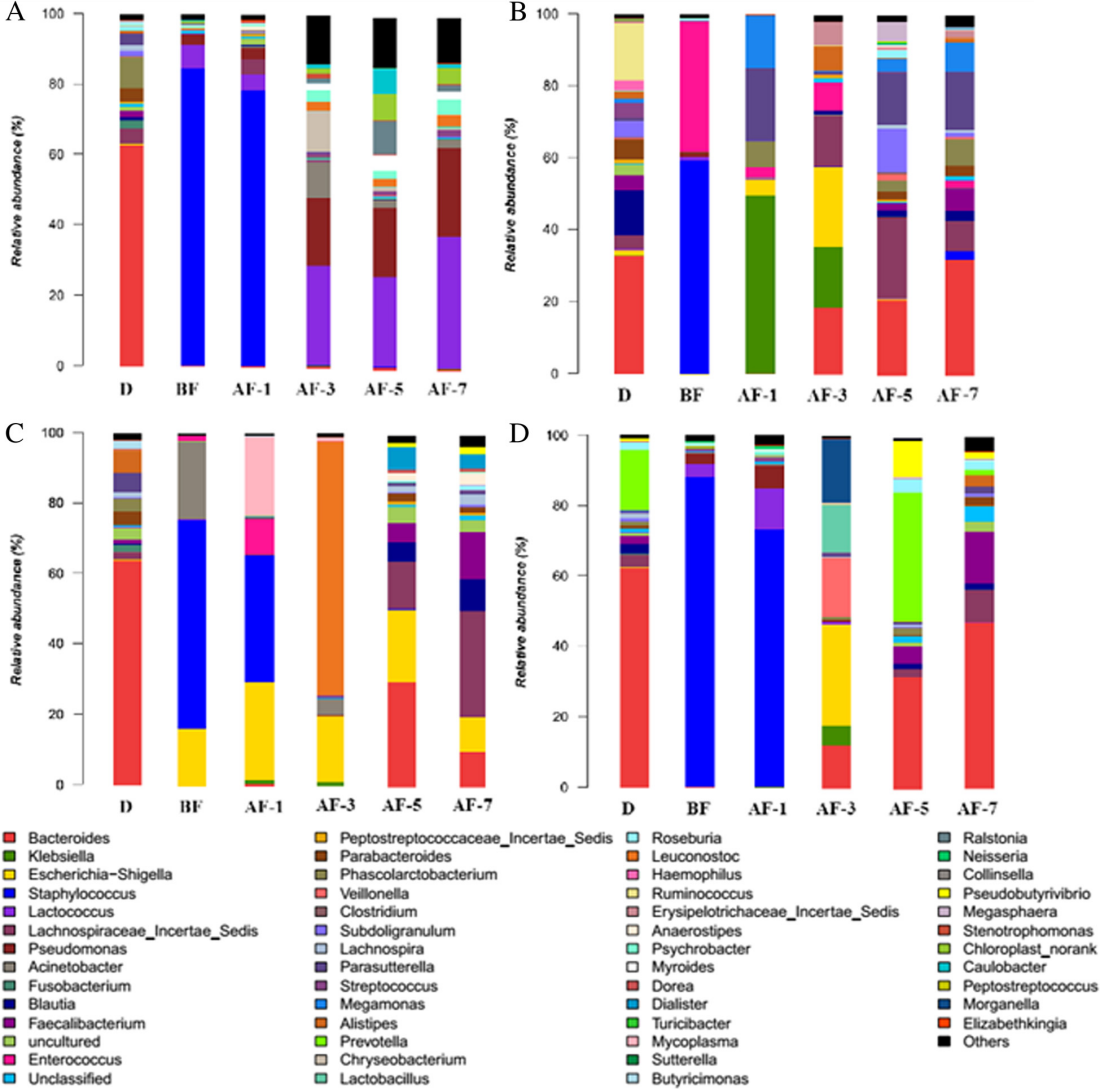
Changes of the flora throughout the course of the disease. D = donor. BF = before fecal microbiota transplantation. AF-1 = one day after fecal microbiota transplantation. AF-3 = three days after fecal microbiota transplantation. AF-5 = five days after fecal microbiota transplantation. AF-7 = seven days after fecal microbiota transplantation. Different colors represent different species

In order to detect the effect of FMT for MSRA enteritis, we collected the stool or ileostomy fluid of patients before and after FMT and stool of the donors to analyze the changes of flora (Fig. 2). We can clearly find from the flora analysis results that all the patients had a decreased intestinal flora species before FMT and the content of staphylococcus aureus almost reached half of total intestinal flora in all patients before transplantation which is consistent with the clinical symptom and examination results. Patients’ gut bacteria after FMT gradually agree with the donors’ reflected the alleviative symptoms.

### Staphylococcal Cassette Chromosome *mec* (SCCmec) Genotype

**Fig. 3:**
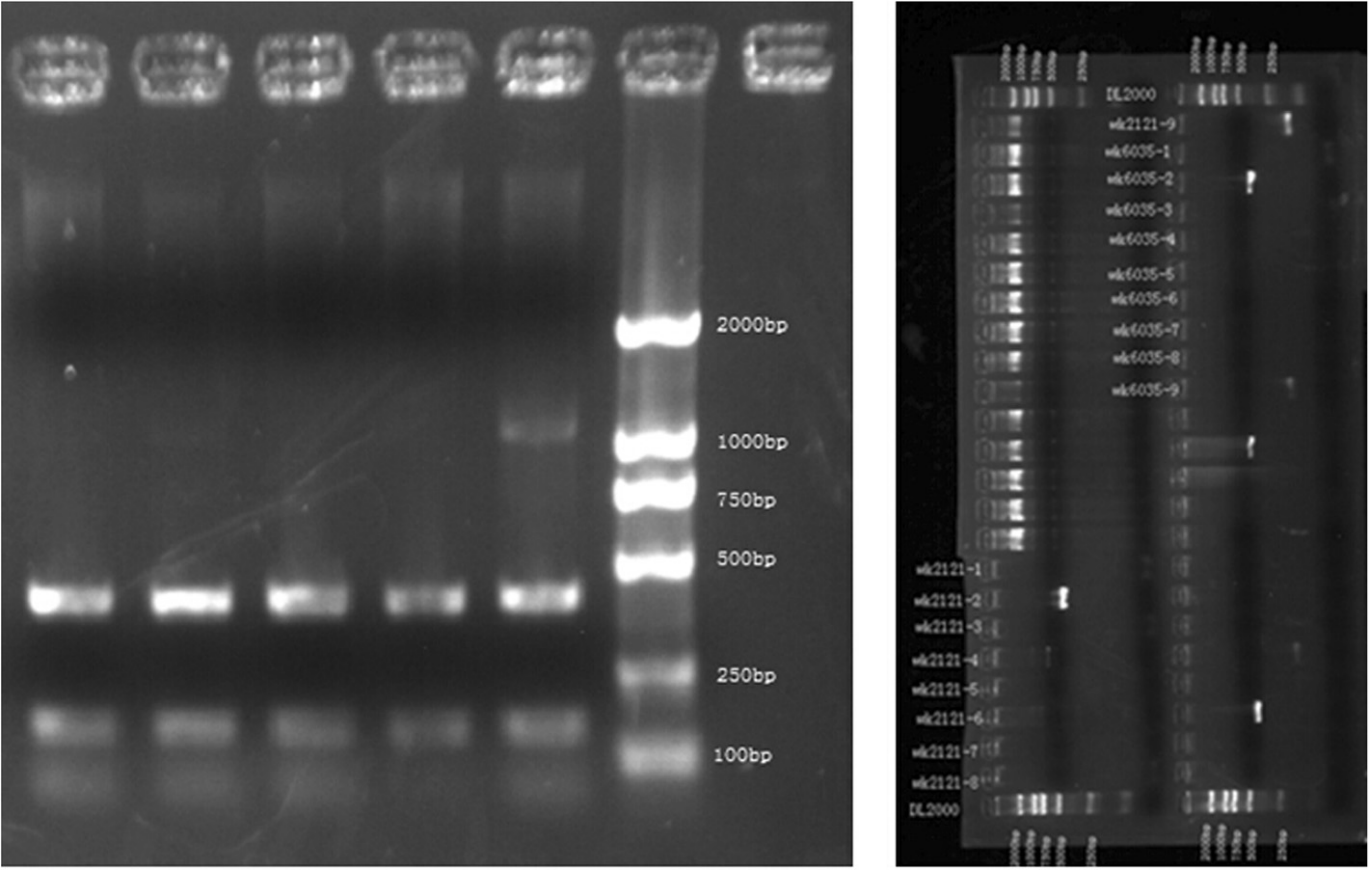
Polymerase Chain Reaction result of Staphylococcal Cassette Chromosome *mec* gene

**Fig. 4:**
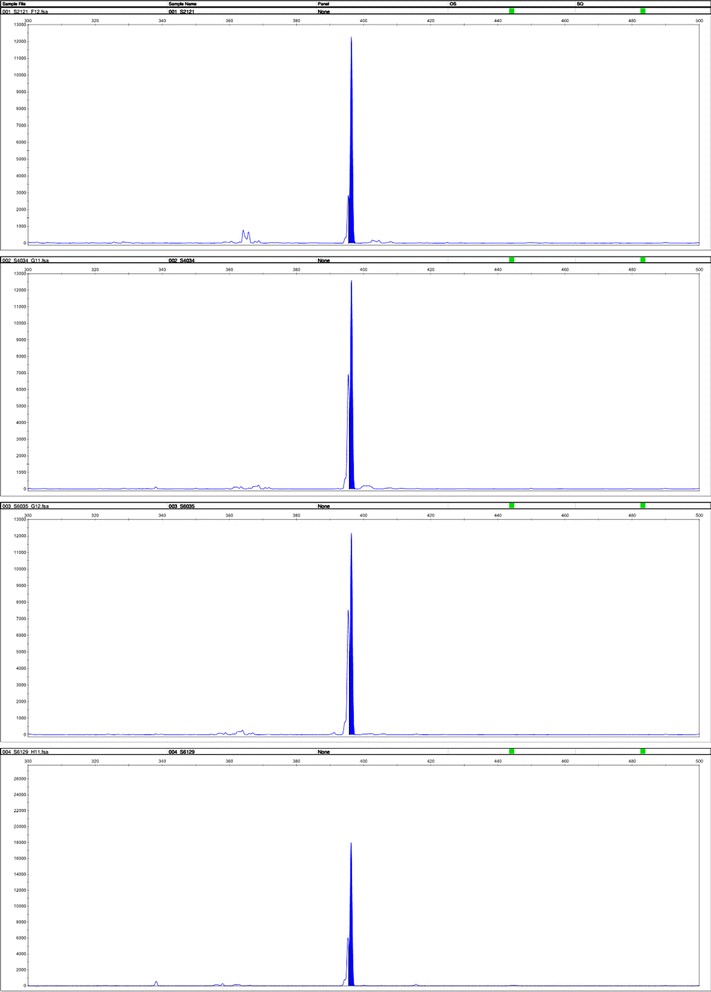
Staphylococcal Cassette Chromosome *mec* Genotype showed the strain of the acquired MRSA is SCC mec typeII as the amplification length are all 398 bp

**Fig. 5:**
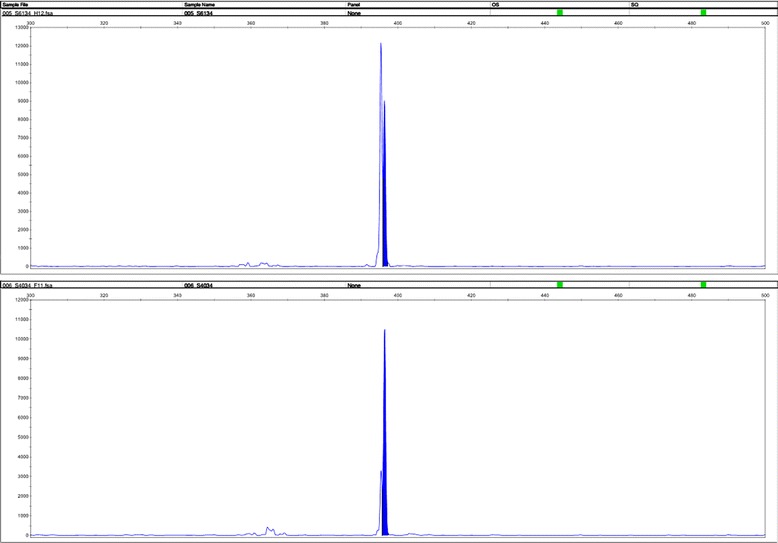
Staphylococcal Cassette Chromosome *mec* Genotype showed the strain of the acquired MRSA is SCC mec typeII as the amplification length are all 398 bp

Totally 6 non-duplicated clinical strains of *S. aureus* isolates from ascites, gastric juice, sputum, ileostomy fluid and feces of the five patients were collected. The genotypes of SCCmec were determined by multiplex PCR. According to the results of STR parting, the length of SCCmec gene amplification in the sample is 398 and make sure that the epidemic strain of this hospital acquired MRSA is SCCmec type II (Figs. 3, 4, 5).

## Discussions

The incidence of antibiotic associated diarrhea is increasing dramatically. About 3 % to 29 % of hospitalized patients suffered from it [6]. Scopetti *et al.* [2] first reported acute enteritis caused by MRSA in 1983. Symptoms of MSRA enteritis usually appear 2 to 7 days after surgery. Patients with delayed clinical symptoms often have normal gastrointestinal peristalsis, leading to fewer toxins within the lumen. MRSA enteritis is characterized clinically by an acute onset in the early postoperative phase of fever, abdominal distension and production of a frothy, brownish-green, mucous watery effluent. The resulting systemic inflammatory response syndrome is associated with a high ileostomy output and related sepsis which can lead to severe dehydration, shock and eventually multi-organ failure even, in some circumstances, death [5, 7, 8].

The human intestine harbors abundant microbiota, which plays crucial roles in our healthy. One important function of it is acting as a barrier against pathogen colonization or overgrowth of resident opportunistic bacteria like MRSA [9]. These processes are made possible when the presence of abundant and diverse microbiota [10]. Disturbances of the microbiota caused by antibiotics have profound effect on its composition and function. But even the normal dose, with indications of anti-infection treatment may result in patients with intestinal flora disturbance like our patients with Crohn's disease because they have a vulnerable intestinal micro ecology and abnormal immune function. In our microbial diversity analysis we found that all the patients had a very low level diversity of the microbiota and *S. aureusis* account for more than half of it. That means sever dysbiosis in the gut leading to sparing MRSA multiply. Compared with MSRA enteritis, enteritis caused by *Clostridium difficile* is more concerned in recent years. This infection is easy to recur and gradually develop resistance to vancomycin. Therefore, fecal bacteria transplantation as the treatment of repeated recurrence CDI has been put forward and obtained excellent curative effect [4]. One of the basic theories of this treatment is intestinal dysbiosis.

The first patient showed up severe diarrhea and hyperpyrexia needing more than 10 L fluid each day at the very start and we had no idea about the reasons, so we use spectrum antibiotic empirically. But it had a poor effect. When we got the result of gastric juice culture telling us a MRSA, we quickly employed Vancocin cp but only find a dissatisfied effect again. Therefore, we tried FMT to rebuild the balance of gut flora which can compete with MRSA as rescue therapy. Subsequently truth from microbiota analysis told us that FMT did rectified the dysbiosis of gut performing at a nearly normal abundant and diverse as the donor and got an excellent curative effect.

Different from the other three Crohn’s patients, JYL was not a patient with Crohn's disease and he is a 19 years boy suffered from congenital intestinal malrotation and Gastroptosis resulting in lots of gastric juice drainage everyday and had been treated with broad-spectrum antibiotic for two months before admitted to our centre, so he himself maybe existed dysbiosis before surgery but we didn’t notice that and MRSA breakout after operation. Generally, antibiotic deplete the overall organism abundance and can also lead to an increase in antibiotic resistant organisms such as *vancomycin-resistant enterococci, methicillin-resistant Staphylococcus aureus* and transfer of antibiotic resistance genes among the microbial community [11]. So FMT seems a more harmless and reasonable measure to treat similar diseases.

However, we still couldn’t tell weather vancomycin or the effective competition of transplanted intestinal flora suppresses MRSA because patients accepted vancomycin at the same time. But at least, we can well-founded finger out that the intestinal flora restored no matter at abundance or diversity and reached a new balance. At the same time, we can see vancomycin did not kill transplanted microbes in a short period of time, but long time effects still needs further study.

## Conclusions

Patients with hospital-acquired diarrhea of unknown etiology, risk factors such as using broad-spectrum antibiotic should alert the physician the possibility of MRSA enteritis. Till now, vancomycin is the first choice treating MSRA enteritis but we here suggest FMT as a first-line measure to cure the dysbiosis coursed by MSRA.

## Abbreviations

MRSA: *Methicillin-resistant Staphylococcus aureus*
FMT: Fecal microbiota transplantation
PPI: Proton pump inhibitors
CRRT: Continuous renal replacement therapy
